# HybridMouse: A Hybrid Convolutional-Recurrent Neural Network-Based Model for Identification of Mouse Ultrasonic Vocalizations

**DOI:** 10.3389/fnbeh.2021.810590

**Published:** 2022-01-25

**Authors:** Yizhaq Goussha, Kfir Bar, Shai Netser, Lior Cohen, Yacov Hel-Or, Shlomo Wagner

**Affiliations:** ^1^Sagol Department of Neurobiology, Faculty of Natural Sciences, University of Haifa, Haifa, Israel; ^2^School of Computer Science, The Interdisciplinary Center, Herzliya, Israel

**Keywords:** animal communication, social interactions, ultrasonic vocalizations, neural networks, machine learning, CNN–convolutional neural networks, LSTM–long short-term memory

## Abstract

Mice use ultrasonic vocalizations (USVs) to convey a variety of socially relevant information. These vocalizations are affected by the sex, age, strain, and emotional state of the emitter and can thus be used to characterize it. Current tools used to detect and analyze murine USVs rely on user input and image processing algorithms to identify USVs, therefore requiring ideal recording environments. More recent tools which utilize convolutional neural networks models to identify vocalization segments perform well above the latter but do not exploit the sequential structure of audio vocalizations. On the other hand, human voice recognition models were made explicitly for audio processing; they incorporate the advantages of CNN models in recurrent models that allow them to capture the sequential nature of the audio. Here we describe the HybridMouse software: an audio analysis tool that combines convolutional (CNN) and recurrent (RNN) neural networks for automatically identifying, labeling, and extracting recorded USVs. Following training on manually labeled audio files recorded in various experimental conditions, HybridMouse outperformed the most commonly used benchmark model utilizing deep-learning tools in accuracy and precision. Moreover, it does not require user input and produces reliable detection and analysis of USVs recorded under harsh experimental conditions. We suggest that HybrideMouse will enhance the analysis of murine USVs and facilitate their use in scientific research.

## Introduction

Many vertebrates use Species-specific vocal communications for social interactions (Todt and Naguib, 2000; Wilkins et al., 2013; Chen and Wiens, 2020). Specifically, mice and rats use ultrasonic vocalizations (USVs) to convey a variety of information types: identification of the emitter and its group, its status within the group and the status of the group, the environmental conditions, such as the presence of predators, and whereabouts of food and water (Lahvis et al., 2011). The emitter and the receiver of the vocal cue can be found at various types of social engagements. For example, pups emit either wriggling calls (WC) to gain their parents' attention or isolation calls following a drop in their body temperature when kept away from their mothers (Haack et al., 1983). An adult male may vocalize to ward off other male intruders or peruse a female in courtship by singing to her (i.e., mating calls, MC), while a female may emit vocalizations to answer her male suiter or to call other females (Neunuebel et al., 2015).

Current tools used to detect and analyze murine USVs rely on user input and classical image processing algorithms to identify and clean USVs, thus requiring manual adjustments and ideal recording environments. More recent tools utilize machine learning and convolutional neural networks (CNN) models (Van Segbroeck et al., 2017; Fonseca et al., 2021) to identify vocalization segments. For example, DeepSqueak (DS), a recent benchmark tool for detecting USVs, relies on neural networks to detect USVs (Coffey et al., 2019). It implements an object detection architecture, namely: regional convolutional neural networks (Faster-RCNN) (Ren et al., 2017). CNN-based models such as DeepSqueak are powerful tools for image processing due to their capability to take advantage of the local spatial coherence of images. In the case of vocalizations, the images are 2D representations of the audio signal transformed to the frequency domain. However, CNN models do not exploit the temporal correlations of audio signals and underperform under noisy recording conditions. On the other hand, recurrent neural networks (RNNs) can compensate for these weaknesses by capturing long contextual dependencies, using prior knowledge, thus offering better performance.

In this study, we describe a USV Extractor software (termed HybridMouse), an audio analysis tool that allows labeling of murine ultrasonic vocalizations both manually or automatically. The automatic detection of USVs is performed by the HybridMouse model, which combines convolutional (CNN) and Bidirectional Long-Short Term Memory (BiLSTM) recurrent neural networks to identify USVs, label them, and extract their denoised representations.

## Methods

### Training a Hybrid CNN-BiLSTM Model to Detect USVs

#### Animals

Mice from three distinct strains: BALBc, C57BL/6J, and CD-1 (ICR) were purchased from Envigo (Rehovot, Israel). The mice were kept in clean plastic chambers (GM500, Tecniplast, Italy) at 22°C and a 12-h light/12-h dark cycle (light on at 7 am) and received food and water *ad libitum*. All cages contained standard wood chip bedding, cotton wool bedding material, and a mock nest of a 12 cm plastic tube. All the recorded adult mice were 3–5 months old, and all the recorded pups were healthy, 3–5 days old pups.

#### Recording the Training Data

Adult vocal communications were recorded using a 1/4 inch microphone (Type 4939-A-011), connected to a preamplifier (Type 2670), and an amplifier (Type 2690-0S1, Bruel and Kjaer) in a custom-built sound-shielded box. Vocalizations were sampled at 250 kHz with a CED Micro 1401-3 recording device (Cambridge Electronic Design Limited, Sunnyvale, CA). The mice were placed in their home cage or a fresh cage during recording sessions and received food and water *ad libitum*. The lid was removed and replaced with a metallic grid to allow passage of sound and prevent escape, while the microphone was placed hovering just above the cage. The system records 10 min every hour for 12 h, during the 1st, 2nd, and 8th nights. In each strain, we recorded four types of pairs: male-male (both naive), female-female (both naive), male-female (both naive), experienced female (dam following weening)-naïve female. In total, we recorded S × G × P × D × H × M = 216 h. When S is the number of strains (3), G is the number of social groups (4), P is the number of pairs in each group (3), D is the number of recorded days (3), H is the number of hours recorded each night (12), and M is the number of minutes sampled every hour (10).

Pup calls were recorded by placing them in a 200 ml cardboard cup and recording for 5 min each. In total, we recorded ten pups per strain from at least two different litters for a total of S × P ×M = 2.5 h, where S is the number of strains (3), P is the number of pups in each group (10), and M is the number of minutes recorded for each pup (5). Even though this group's recorded hours are low, we extracted ~4k pups isolation USVs. This is because isolated pups emit vocalizations rigorously as their temperature drops almost throughout the entire (5 min) recordings, resulting in a high number of USVs per recording, while other groups tend to vocalize fewer hours. As a comparison, we also recorded interactions between naïve male dyads, and we did not use these recordings in our training because the number of USVs emitted was too low.

#### Dealing With Imbalanced Data

Only 0.44% of the data contained USVs (57 min of 216 h recorded), making the data highly imbalanced and unsuitable for a classification task. To overcome this, we trained the data only on short (1–20 s) audio segments containing consecutive USVs with short (<1 s) intervals between them, which effectively condensed our training data from ~200 h to ~2 h with more balanced training examples (45% USV and 55% background).

#### Noise Samples

Condensing the data meant that we trained the model only on the background noise between the USVs, which posed a new problem; it was insufficient to adequately train the model, resulting in a high rate of false-positive events. To overcome this problem, we randomly sampled noise segments from our recordings and added them to the training data. We also added background recordings made in other labs to diversify noise patterns and thus generalize the model. In total, we added 2600 files (1-s long each) without USVs, for a total of ~43 min.

To overcome the imbalance reintroduced to our data due to adding noise samples, we used a weighted classifier in our training to represent the imbalance in the data between the two classes (USV 25%, background 75%).

#### Manual Labeling

A trained team of analytics manually identified each USV by screening spectrograms of all the recorded data using a custom-made Matlab script. When labeling the USVs, our main priority was to extract temporal features (such as syllable duration, call duration, syllable rate per sentence and second, etc.). For that reason and to save time and effort, we only extracted the beginning and ending timestamps of each USV, which means that the minimal and maximal frequencies for each USV were not extracted.

### Evaluation

#### Evaluation Metrics

Model evaluation was made by comparing the model predictions to manually labeled data and extracting recall, precision, and *F*1 scores, as follows:


$$\begin{array}{r} Recall=\;\frac{True\;Positive}{True\;Positive+False\;Negative}\\ Precision=\;\frac{True\;Positive}{True\;Positive+False\;Positive}\\ F1\;=2^{*}\frac{Presicion^{*}Recall}{Presicion+\;Recall} \end{array}$$

Where

*True-positive*—The number of correctly identified USVs

*False-positive*—The number of noise segments mislabeled as USVs 

*False-negative*—The number of USVs the were not detected.

#### Local and Global Signal to Noise Ratio Calculations

The magnitude of an audio segment was calculated as the mean sum of squares of the raw audio values. To calculate the local signal-to-noise ratio (SNR), we first calculated M_sig+bg,i_, the magnitude of the ith audio segment containing USV. We then calculated M_bg,i_, the mean magnitude of the audio segments preceding and following the i^th^ USV segment (that do not contain USVs). Then we calculate R_i_, the local SNR of the i^th^ USV, as follows:


$$\begin{array}{l} R_{i}=\;\frac{M_{sig+bg,i}-M_{bg,i}}{M_{bg,i}} \end{array}$$

Where

*R*_*i*_–The local SNR of USV segment *i*

*M*_*sig*+*bg,i*_–The magnitude of the audio segment containing USV segment *i*

*M*_*bg,i*_–The magnitude of the audio segments preceding and following USV segment i.

To calculate the global SNR of the entire audio file, we calculated M_sig+bg_, the magnitude of all segments containing USVs combined, and M_bg_, the magnitude of all the segments not containing USVs combined, and repeated the calculation above.

#### Testing Data and Labeling

The analysis was performed on audio files downloaded from the mouseTube database (https://mousetube.pasteur.fr/). The files were recorded by two laboratories during various social tasks to ensure model generalization. Files from the first laboratory (Duke University Medical Center, Social context comparisons) included recordings of adult male B6D2F1/J mice with either awake or anesthetized male or female conspecifics on different menstrual states or only with urine from males or females. Files from the second laboratory (Washington University in St. Louis, Pup USV Day 3–14 Monitoring) included recordings of C57BL/6J pups (p3–p14). All recordings were made using Avisoft CM16/CMPA microphones at a 250 kHz sampling rate (for more details, see Supplementary Table 1). USVs detected by the different models were labeled as “USV.” Subsequent tagging of the same USVs by a model were labeled “Partial,” and single taggings encompassing multiple USVs were labeled “Multi.” Each of these labels was counted only once. In addition, tagged alarm calls were labeled as “AC” and excluded from further analysis.

#### Comparison With DeepSqueak

We analyzed the same test files using DeepSqueak (Coffey et al., 2019), employing its default network for mouse USVs. We performed the analysis with two different settings; balanced (DS_B) and high recall (DS_H); all other settings were kept unchanged in their default settings: Total analysis length = 0 (full duration), Analysis chunk length = 3 s, Overlap = 0.1 s, frequency cut off high = 120 kHz, frequency cut off low = 1 kHz, score threshold = 0. The analysis was performed using the built-in “Mouse Call_Network_V2” network.

#### Statistical Analysis

Model performances were compared using the Mann-Whitney U test due to the non-normality of the variables. The analysis was performed using SPSS version 27 software.

### Our Model

#### Model Architecture and Syllable Isolation

As mentioned in the methods (see section Manual Labeling), we labeled the recorded data by manually extracting the start and end time points of each USV and not their frequency boundaries. Therefore, the model output contained only temporal data (each USV's start and end time points) and no spectral data (such as USV frequency and structure). However, our goal was to create a model capable of not only identifying the time steps containing USVs but also of estimating their frequencies and segmenting them from the background noise. To achieve this, the model processes the 124 by 51 feature maps in two phases; CNN and BiLSTM. The output of the CNN phase is a 1×124 vector estimating the probability of each frequency to be a part of a USV. Concatenating these vectors along with the temporal domain into a 2D map allowed us to use it as a mask to clean the raw spectrograms (**Figure 2B**, Classification and mask). In the second phase, the BiLSTM layer classifies each vector (representing each time point) and estimates whether this vector is part of a USV or not.

##### CNN Layers

**Figure 1:**
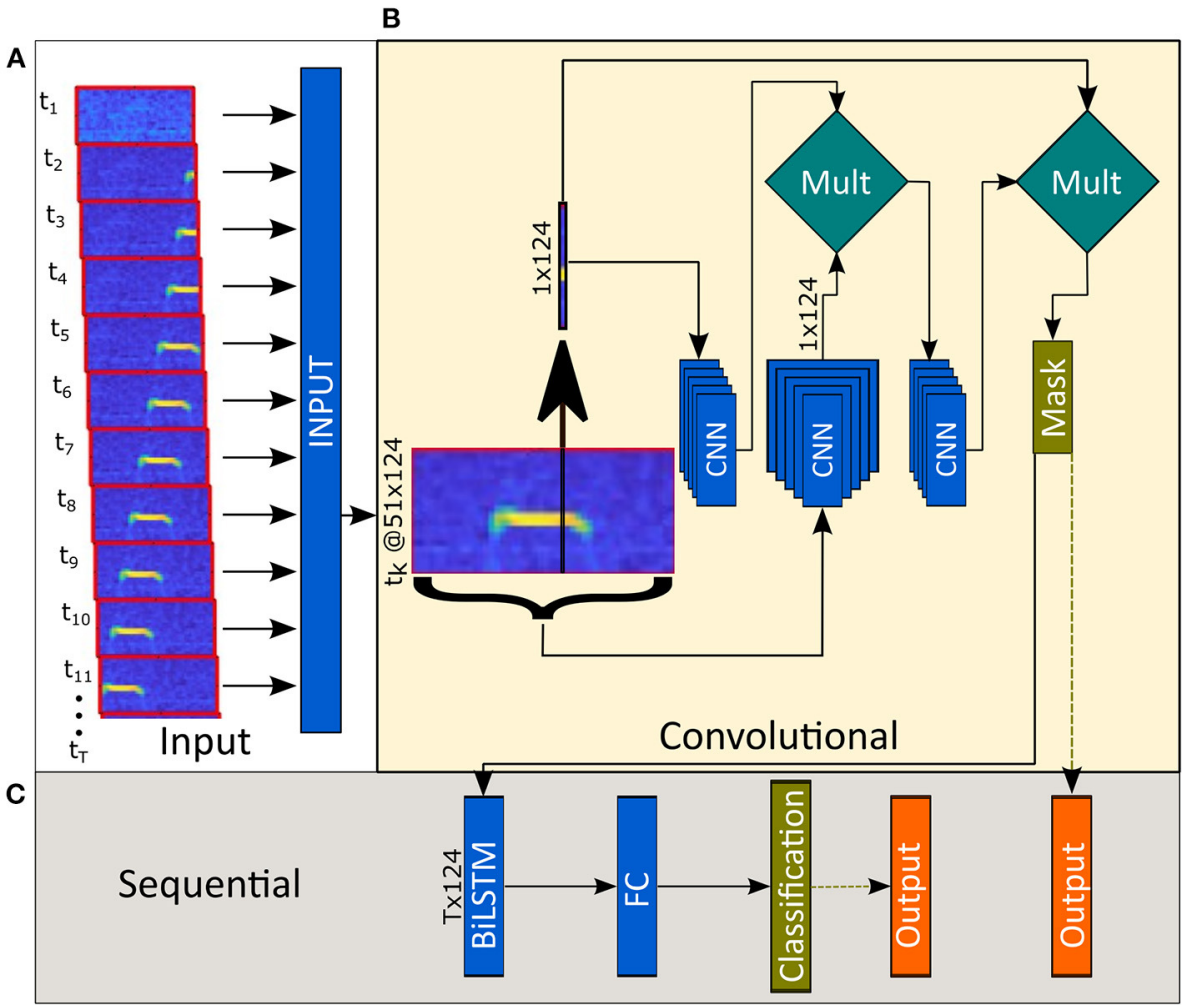
Model architecture. **(A)** Sequential data (124 by 51 Mel-spectrograms per time step) are fed into the model. **(B)** The first phase consists of convolutional layers and outputs a mask defining the estimated frequency (or the lack of) of USVs. **(C)** The second half of the model combines data from all time points and classifies each time step.

**Figure 2:**
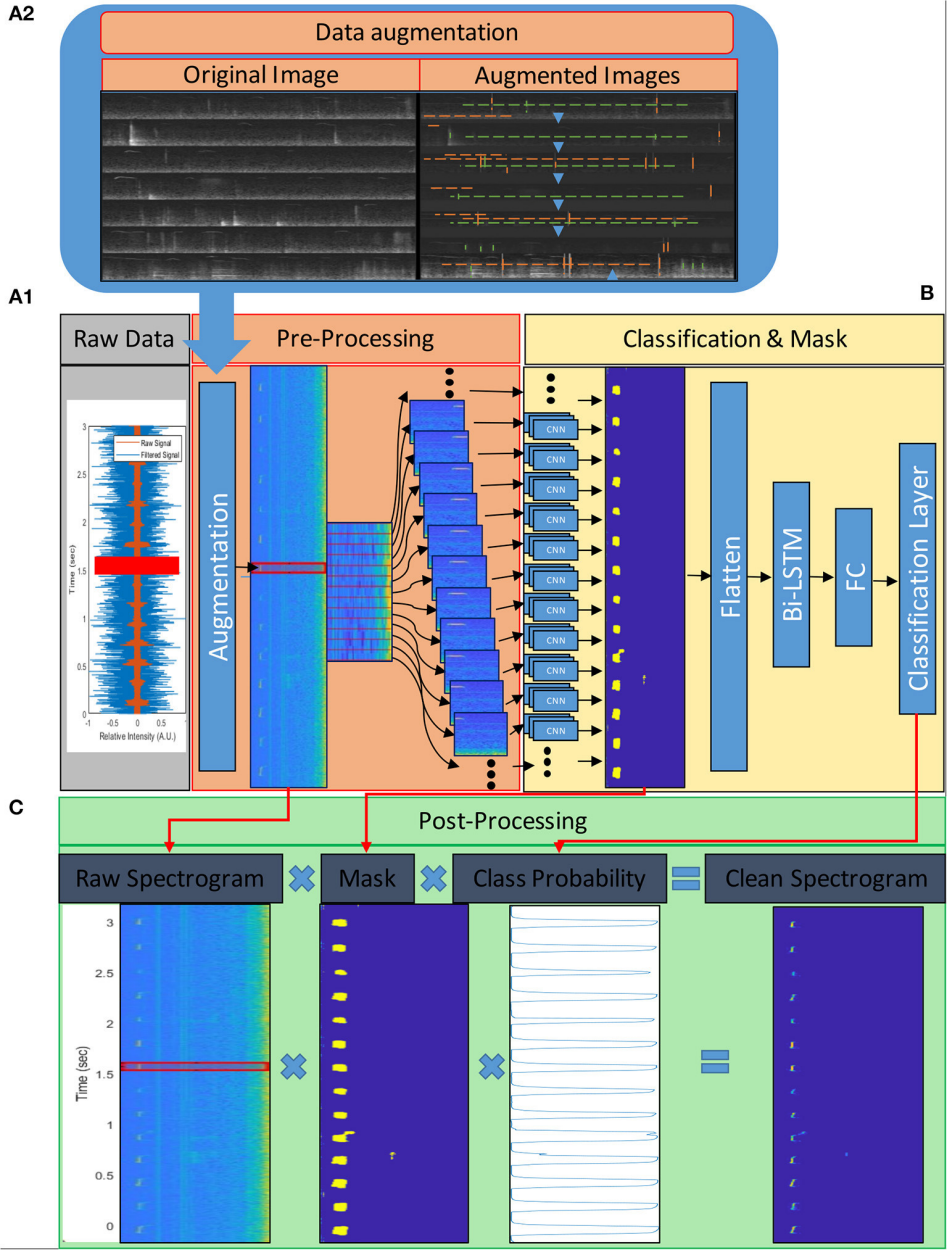
USV processing flow chart. The vocalizations are recorded at a high sampling rate *(*>*250 samples per sec)* and, after scaling, undergo processing, which can be divided into three phases; **(A)** pre-processing, **(B)** syllable detection, and **(C)** post-processing. **(A)** First, the signal is converted to the frequency domain by extracting Mel-frequency cepstrum (MFC) features; the spectrograms indicate frequency contours and relative intensities of the frequency components. The extracted frequencies are binned in 124 frequency bands (range 1–125 kHz) and 332-time steps per second (~3 ms per time step); **(A.1)** These frequency maps are augmented during training to create a more generalized model; the insert **(A2)** shows spectrograms of training images before (Original) and after (Augmented) augmentation. Blue arrows indicate the vertical translation direction of the images; green dashed lines indicate existing noises' location; orange dashed lines indicate randomly added lines to simulate humming (horizontal lines) and impulse (vertical lines) noises. We also scaled the values of the images in the training data but did not do so in this example to make it easier to plot. Each time step is fed into the classification model along with its preceding and following 25-time bins. **(B)** The 124 by 51 feature maps are processed by the model in two phases; CNN and BiLSTM. The CNN layers filter the feature maps and produce 124 by 1 column masks for each time step. These masks indicate the presence (or lack of) and frequency of USVs in their respective time steps. The masks are flattened and fed into the BiLSTM layers and to fully connected layers (FC) to classify each time step as a “1” or “0,” indicating whether they contain/are part of a USV or not, respectively. The output of these layers is also used in **(C)** the post-processing phase to produce a cleaned signal. The cleaning is done by multiplying the original spectrogram with the mask and the probability vector produced by the classification model.

Each time bin is fed into the model along with its neighboring 25-time steps. We experimentally found this number of neighboring steps to be the minimum number that yields an optimal performance of the model. While models with fewer neighbors resulted in inadequate predictive capabilities, models with a higher number of neighbors did not improve the model enough to justify increasing complexity. Inputting each time bin with neighboring 25-time steps creates a form of self-attention mechanism (Vaswani et al., 2017; Bello et al., 2019); a 1D convolution is performed on the mid-time bin to accentuate potential USVs, and a 2D convolution is performed on the entire feature map to suppress global noises (Figure 1). By multiplying the outputs of the two branches, we got a clean 1D representation of each time-bin. Finally, we multiplied the input by the clean 1D vectors before passing it to the sequential layers to force it to act as a filter. The output of this phase is used again in the post-processing stage to produce clean syllables Figure 2C (see section Pre-processing and Augmentation).

##### Sequential Layers

The extracted vectors from the CNN layers are first reshaped, i.e., the vector is transformed from a 124×1×1 vector (Height x Width x Channel) into a 1×124 (1 × Channel) vector. They are then fed into the sequential layers, comprised of BiLSTM layers followed by fully connected (FC) layers. Finally, a weighted softmax-cross entropy algorithm was implemented in the classification layer to output a categorical 1D vector where each time step was labeled either “0” or “1” for “background” and “USV,” respectively. It is also possible to output a numeric 2D probability vector with values between 0-1 for each label (“background” or “USV”) at each time step. The output is the model's estimated probability of each time step to be part of a USV (Figure 1C). The model was trained using 1-s long audio segments (332-time steps). However, it is possible to feed longer or shorter longer segments to train the model or to extract USVs.

#### Training Data

Training data was acquired by recording vocalizations emitted during dyadic interactions between mice of various strains, ages, and during various social contexts. In total, we recorded over 200 h of mouse vocal communications representing over 1,200 h of interactions. We then meticulously combed the audio recordings and extracted over 40k USVs to be used as training data (See methods for details).

The training data included two types of audio clips; (1) files containing “calls” (a series of USV syllables) and (2) files containing only background with no calls. Most of the background samples were extracted from the recorded training data, but some samples were extracted from recordings made by other teams and laboratories in order to diversify the noise parameters and make the model suitable for general recording conditions.

It is important to note that the model was tested and compared to DeepSqueak on an entirely new set of files downloaded from mouseTube. The recordings that were used to train the model, both the ones recorded at our lab and those collected from others, were not used to test the model. The files used for testing were recorded under different conditions at different laboratories.

#### Retraining and Fine-Tuning

We added the option to retrain the model. This can be performed by manually labeling or automatically labeling (and manually adjusting) samples of recordings and retraining the model to create new, customized models.

#### Pre-processing and Augmentation

For pre-processing of audio clips (Figure 2A), the signal was converted to the frequency domain by extracting Mel-frequency cepstrum (MFC) features. The extracted frequencies were binned into 124 frequency bands (range 1–125 kHz) and 332-time steps per second (3 ms per time step). Each time step was fed into the classification model along with its preceding and following 25-time steps (bins) as 124 by 51 images for each time step (Figure 2A). We also converted the labels' timetables into the corresponding one-dimensional categorical vectors. Each label vector had the number of time steps as the corresponding feature map, and each time step that contained a part of a USVs was labeled “1,” and the rest that did not contain parts of a USV were labeled“0.”

We have used a similar pre-processing of audio clips for training and testing, aside from the following augmentations. During training, the images were augmented in three ways. USVs vary markedly, depending on the emitter's strain, genotype, age, sex, socio-emotional state, etc. Although our training data included a great variety, it did not include all types of mouse USVs. Therefore, we randomly shifted the feature maps along the feature-axis (frequency axis) in order to train the model to recognize new vocalizations emitted at different frequency bands while preserving their basic structure. In addition, the scale of the signal depends on the acquisition system and method and, ideally, should be normalized (zero-centered and with a range between −1 and 1). Signal normalization can be performed by subtracting the mean from the signal and dividing it by the maximum value. However, since the audio signal is fed in short segments, it was challenging to shift and scale it that way. In addition, normalizing the feature maps was also not ideal due to the non-linear nature of the Mel-spectrogram transformation. To overcome these difficulties, we randomly scaled (added values [−3, +3 a.u]) and shifted (vertical shift [−50,+10 a.u]) the feature maps during training and trained the model to be less sensitive to the input scale and center. Lastly, horizontal and vertical lines were randomly added to simulate continuous humming and impulse noises, respectively (Figure 2A.1). We also tried other, more traditional augmentation methods, such as adding Gaussian or pepper-and-salt noise to the images. In these cases, however, when the augmentation levels were high, the model accuracy declined, and when the levels were low, the augmentations didn't have an effect; therefore, we did not use these augmentations.

#### Post-processing

The last step of USVs extraction took place after the model prediction. The output of the convolutional layer was used as a mask and multiplied with the raw spectrogram and the probability vector to extract clean syllables without background (Figure 2B).

#### The HybridMouse Software

We integrated the HybridMouse model in a user-friendly Matlab app (Supplementary Figure 1). This app was designed to facilitate comparisons between recordings from up to two microphones. Our original paradigm includes recording from one main microphone (Main Mic) and an additional microphone (Mini Mic).

## Results

### Syllable Isolaion and Denoising

**Figure 3:**
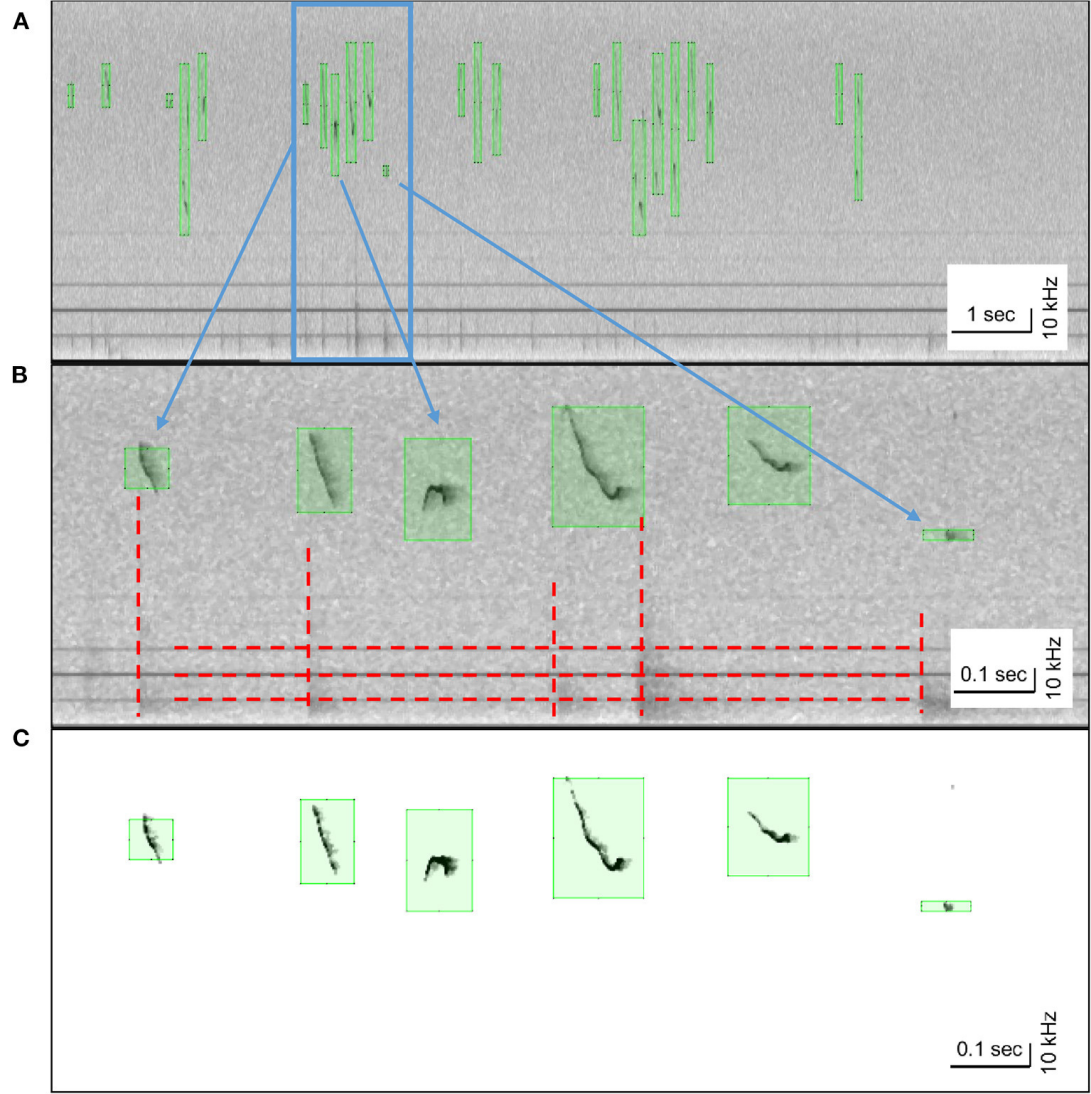
USV detection and denoising. Spectrograms (linear) of example USVs detected by HM model. **(A)** Detected USVs over a 10-s audio file; green squares indicate the estimated frequency and time boundaries of each USV. **(B)** A close-up of the highlighted blue area in **(A)** containing both types of noises, humming, and impulse noises, denoted by vertical and horizontal red dashed lines (respectively). **(C)** The denoised version of the USVs shown in **(B)**, processed by HybrideMouse; all background noises were removed, and the USVs were isolated.

HybridMouse model extracts the timestamps of each USV, its frequency, and a clean denoised representation. These capabilities were tested on 58 audio files (180–300 s total length each) downloaded from the Mousetube database and recorded under different conditions and settings. We found that the model excels in estimating the onsets and endings of the syllables (Figures 3A,B). We also found that the model manages to produce denoised representations of the USVs (Figure 3C), which can be used to extract additional features such as USVs' frequency boundaries and more.

### Comparing Performance of HybridMouse to DeepSqueak

**Figure 4:**
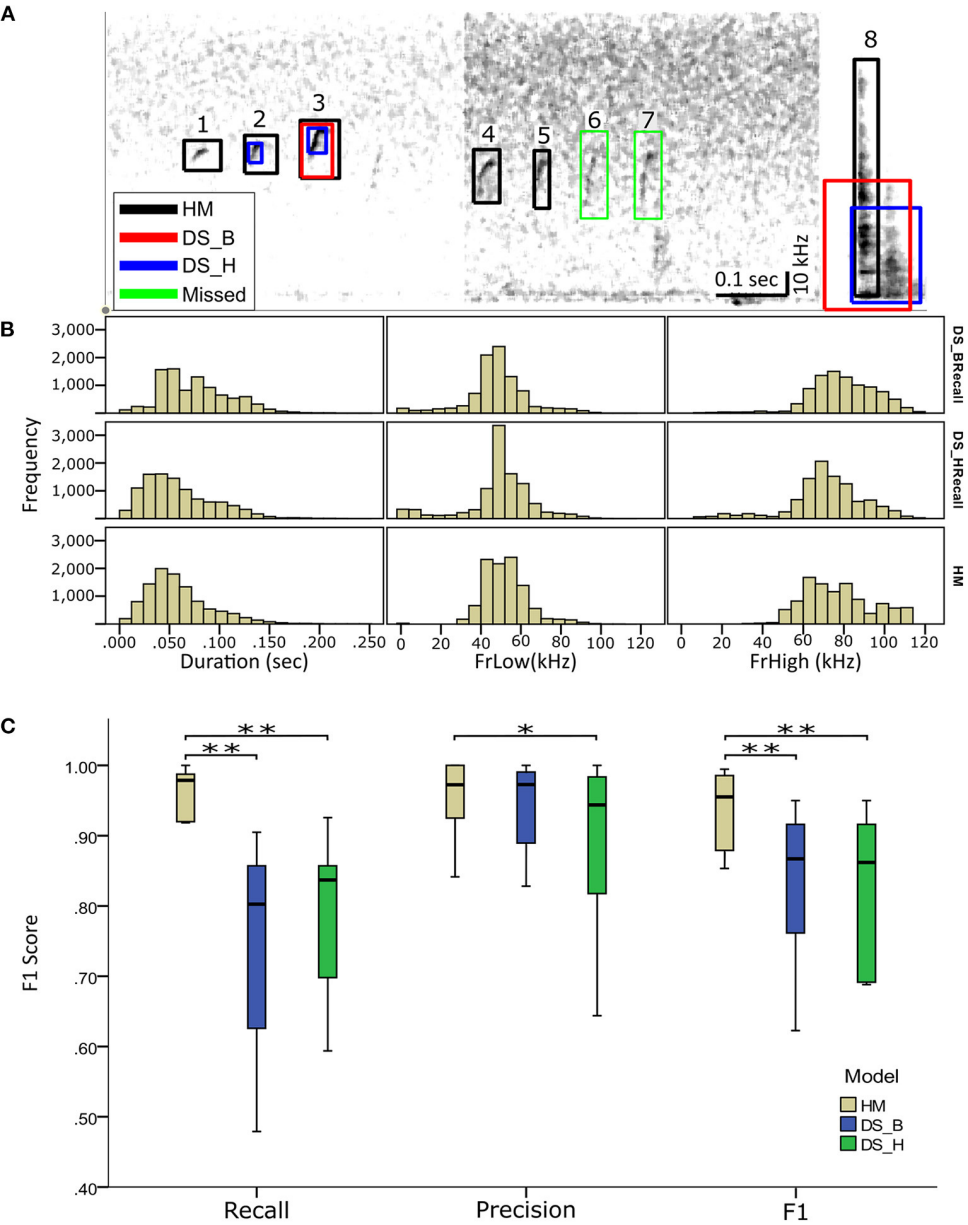
Comparing detection of USVs by HybridMouse to DeepSqueak. **(A)** Visual comparison between the models and examples of their detection performance, showing spectrograms (linear) of audio segments with the detected USV markings by all models; HM (black), DS_H (blue), and DS_B (red) and the missed USVs (green). Shown are USVs detected by all models and settings (3), USVs detected only by HM and DS in high-recall settings (2), USVs detected only by HM (1, 4, and 5). We also see USVs missed by all models (6 and 7) and finally, a noise segment that all models mislabeled as a USV (8). **(B)** Comparing temporal-spectral estimations; Audio files were analyzed using HM and DeepSqueak with balanced recall (DS_B) and high recall (DS_H) settings. Each detected USV's duration, lower-frequency (FrLow), and upper-frequency (FrHigh) boundaries were estimated and compared. **(C)** Comparing the performance of the HybridMouse model (HM–blue) to the DeepSqueak model with two different settings; balanced recall (DS_B–green) and high recall (DS_H–yellow). Wilcoxon signed-rank *p < 0.05, **p < 0.01. HybridMouse scored significantly higher in recall (Mdn = 0.979) than DeepSqeak on balanced recall setting (Mdn = 0.802) (Wilcoxon signed-rank *z* = −2.666, *p* < 0.01), and on high recall setting (Mdn = 0.837) (Wilcoxon signed-rank *z* = −2.666, *p* < 0.01). All models performed rather similarly in the precision, with DS_H receiving the lowest score. HybridMouse achieved significantly higher *F*1 score (Mdn = 0.955) than DS_B (Mdn = 0.876) (Wilcoxon signed-rank *z* = −2.66, *p* < 0.01), and DS_H (Mdn = 0.862) (Wilcoxon signed-rank *z* = −2.66, *p* < 0.01).

We compared the performance of HybridMouse to DeepSqueak, a benchmark model for rodent USV detection (Coffey et al., 2019). To that end, we analyzed the same files using DeepSqueak with two different settings; balanced recall (DS_B) and high recall (DS_H) (Table 1). We found that most of the USVs were detected by both models with high accuracy, and, naturally, the detection capabilities declined in noisier sections. Yet, in most cases, the detection capabilities of HybridMouse were higher than DeepSqueak in both settings (Figure 4A).

**Table 1:** Comparing temporal-spectral estimations.

	**HybridMouse**	**DeepSqueak balanced recall**	**DeepSqueak high recall**
Total number of USVs	10,327	9,175	10,091
Duration (Mdn, sec)	0.052	0.072	0.0512
FrLow (Mdn, kHz)	50.6	47.7	50.1
FrHigh (Mdn, kHz)	73	78.8	71.4
FrLow (<20 kHz) (%)	0.3	8.8	10.7
FrHigh (<20 kHz) (%)	0	1.2	4.3

*Duration—the estimated median length of the USVs in seconds, FrLow and FrHigh—the estimated median lower and upper (respectively) frequency boundary of the USV in kHz, FrLow (<20 kHz), and FrHigh (<20 kHz)—the percent of USVs that were estimated to have lower or upper (respectively) frequency boundaries lower than 20 kHz*.

Using all models, we estimated the detected USVs duration as well as the lower (LowFr) and higher (HighFr)-frequency boundaries. We found that the estimations of frequency boundaries (low and high) and USV lengths were similar between all models. However, the frequencies estimated by DeepSqueak contained more USVs with frequencies lower than 20 kHz (Table 1; Figure 4B; Supplementary Figure 2). These USVs could have been USVs that DeepSqueak was able to detect but could not accurately estimate their frequencies or low-frequency noise segments that it mislabeled as USVs. These results suggest that DeepSqueak has lower precision due to a larger number of false-positive events, especially in high-recall settings, compared to HybrideMouse.

On the other hand, using a lower threshold enabled DeepSqueak, when set on high-recall, to capture the duration of the USVs more reliably. The median value estimated by this setting is more similar to the value that HybridMouse estimated. This was in contrast to when DeepSqueak was set to a balanced-recall setting, which only registered the longer USVs and ignored the shorter ones. Notably, HybridMouse suffers from low accuracy when estimating the higher frequency boundaries due to the spectral map's representation as a Mel-spectrogram with higher resolution in the lower frequencies than the higher ones.

To quantify the detection capabilities of HybridMouse and statistically compare it to DeepSqueak, we manually labeled 13 arbitrarily selected audio files and extracted each model's precision, recall, and *F*1 score. Of these 13 files, four contained very few USVs (~2) and were excluded from the analysis (Table 2; Supplementary Tables 1–3). As expected, running DeepSqueak on a high-recall setting missed fewer USVs, thus achieving higher recall than a balanced-recall setting; however, this comes with the price of mislabeling more noise fragments as USVs and performing poorly on the precision metric. On the other hand, HybridMouse missed fewer USVs and mislabeled fewer noise fragments as USVs than both DeepSqueak settings and in all the tested files. HybridMouse scored significantly higher in recall (Mdn = 0.979) than DeepSqeak on balanced recall setting (Mdn = 0.802) (Wilcoxon signed-rank z = −2.666, *p* < 0.01), and on high recall setting (Mdn = 0.837) (Wilcoxon signed-rank z = −2.666, *p* < 0.01). All models performed similarly in the precision, with DS_H receiving the lowest score. However, the difference in recall was reflected in the final *F*1 score with HybridMouse achieving significantly higher score (Mdn = 0.955) than DS_B (Mdn = 0.876) (Wilcoxon signed-rank z = −2.66, *p* < 0.01), and DS_H (Mdn = 0.862) (Wilcoxon signed-rank z = −2.66, *p* < 0.01). Thus, HybridMouse outperformed DeepSqueak (Figure 4C).

**Table 2:** Comparing detection of USVs by HybridMouse to DeepSqueak.

	**HybridMouse**	**DeepSqueak balanced recall**	**DeepSqueak high recall**
Recall	0.98	0.80	0.84
Precision	0.97	0.97	0.94
*F*1	0.96	0.87	0.86

*Comparing median recall, precision, and F1 scores of the HybridMouse model to the DeepSqueak model with two different settings; balanced recall and high recall*.

**Figure 5:**
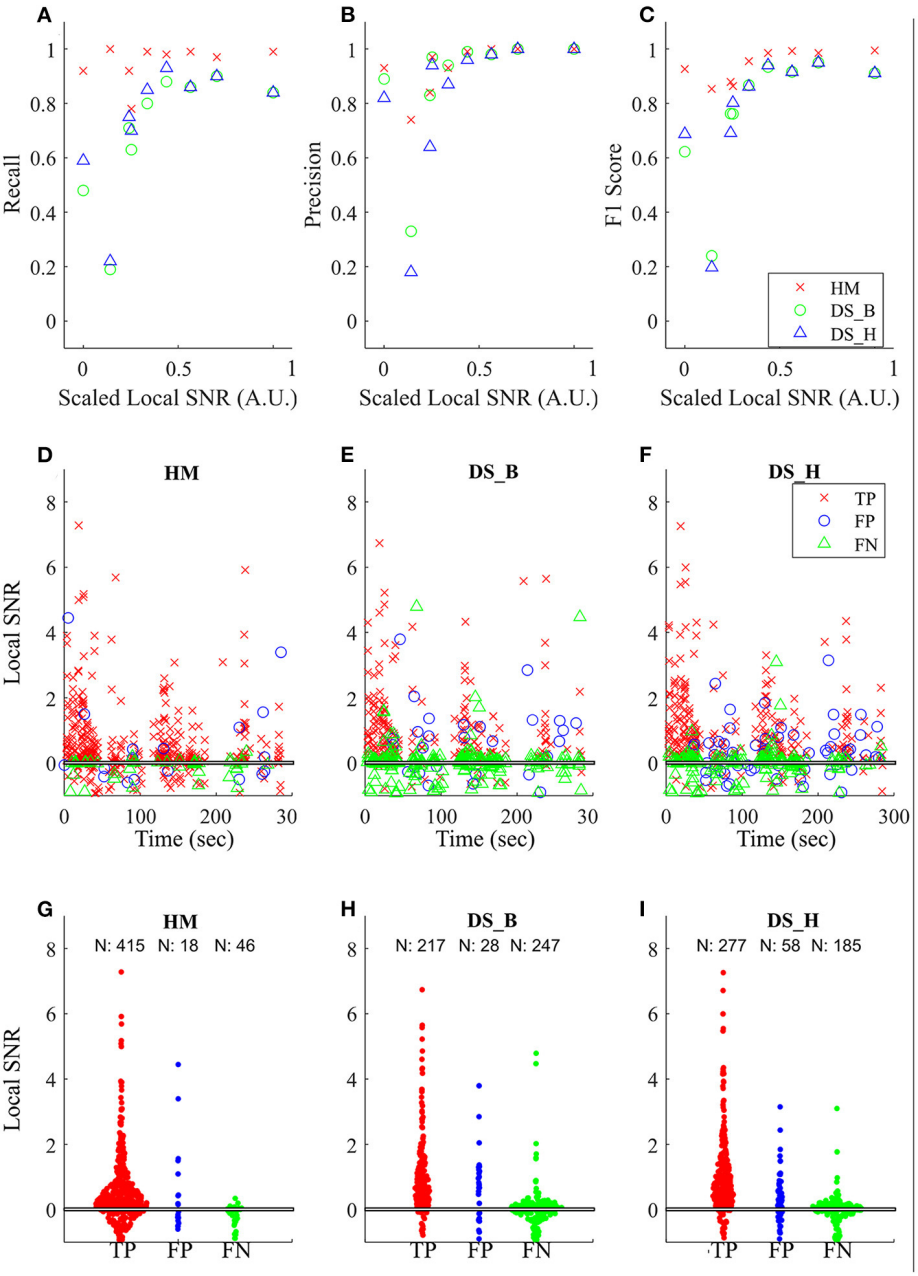
Comparing the effects of SNR on detection. Comparing the effects of SNR on detection capabilities of the model using the three parameters **(A)** recall, **(B)** precision, and **(C)** *F*1 score. The relative SNRs were calculated as described in the methods and rescaled [0,1]. The performance of HybridMouse (HM) remains relatively high. In contrast, the performance of DeepSqueak in both settings (balanced and high recall) (DS_B and DS_H, respectively) drops in all parameters when the relative SNR is low. The middle and lower panels show an example of one of the tested files (interaction between male and female, 300 s duration). The black horizontal line (overlapping with the x-axis) indicates the point where the magnitude of the signal is equal to that of its surroundings. The middle panels **(D–F)** show the detected USVs (TP), missed USVs (FN), and segments mislabelled as USVs (FN) by the models throughout the interaction. And the lower panels **(G–I)** show swarm plots of these parameters and the number of detections in each category.

Lastly, to show that our model is more robust to low SNR, we calculated the global SNR of each tested audio file and found that HybridMouse performs well even in low SNR (Figures 5A–C). This is further demonstrated by the example of one of the tested files (interaction between male and female, 300 s duration shown in Figures 5D–I), showing that below 0.1 local SNR, DeepSqueak struggles to detect the USVs, resulting in a high number of false-negative events. Though lowering the detection threshold (high recall) improves the detection (fewer false-negative events), it comes at the cost of raising the number of false-positive events. In addition, we compared the power spectral densities of the detected USVs and the missed USVs by HybridMouse (Supplementary Figure 3) and again showed that the missed USVs have low amplitude and low SNR.

## Discussion

Like many vertebrates, mice convey various information through vocal communication (Todt and Naguib, 2000; Wilkins et al., 2013; Chen and Wiens, 2020). For these reasons, analyzing social vocalizations can progress our understanding of animal social behaviors and the underlining mechanisms governing these behaviors. Studies in this field rely heavily on observations and subjective assessments based on visual cues, such as freezing and body pose. However, these studies tend to ignore the vocal cues, which can be paramount to understanding and accurately assessing the socio-emotional state of the animals.

One of the reasons this field is ignored is that most murine vocalizations are ultrasonic and inaudible to the human ear and can also be very short and sparse. Another reason is the shortage of tools that may be used for automatic high-throughput analysis of such vocalizations.

With the recent explosion of computerized algorithms and the rise of computational power, multiple tools have been developed to analyze animal vocal communication, such as VoICE (Burkett et al., 2015), MUPET (Van Segbroeck et al., 2017), which rely on classical signal and image processing methods. More recent models such as USVSEG (Tachibana et al., 2020) and Acoustilytix (Burkett et al., 2015) rely on machine learning and deep learning tools to extract useful information from the recording of animal vocalizations. One of the most popular and publically-available models for rodent USV analysis is DeepSqueak, a benchmark in this field that outperforms its predecessors (Coffey et al., 2019). This model relies on deep neural networks to extract USVs from audio recordings. However, DeepSqueak relies only on convolutional neural networks. These CNN-based object detection models such as YOLO (Redmon et al., 2016) and Faster R-CNN (on which DeepSquak is based) perform well under ideal recording conditions; however, they are not suitable for analyzing recordings with a low signal-to-noise ratio. This is because they are made explicitly for image analysis and are less optimized for auditory data, which is represented by a sequence of images rather than a single image; thus, these models do not allow capturing the syllables within their context. Other models based on recurrent neural network-based models are more suitable for analyzing data that have sequential nature.

In this study, we presented HybridMouse, a hybrid CNN-BiLSTM based model for mouse USV extraction. This hybrid model incorporates both CNN and recurrent neural networks (BiLSTM), thus exploiting both the auditory signal's structure and its context.

We decided to compare HybridMouse to DeepSqueak, which is considered a benchmark model for analyzing murine USVs; it is well-known and free, unlike other available tools. DeepSqueak comes with four different pre-trained models, and the model chosen for comparison was trained explicitly for mouse USVs, with two different settings: balanced recall (DS_B) and high recall (DS_H). All other parameters were kept at default settings.

The HybridMouse model was tested on novel data recorded in different labs on which it was not trained and was found to be a robust tool for automatically identifying and isolating USVs even in harsh recording conditions with low SNR. We found that HybridMouse manages to detect almost all of the manually identified vocalizations, resulting in high recall without increasing the rate of false-positive events, thus demonstrating high precision. We compared HybridMouse to DeepSqueak in a balanced setting. We found that although the precision of both models was similar, DS_B identifies fewer USVs, resulting in a significantly lower recall and an overall lower *F*1 score. We also compared HybridMouse to DeepSqueak in a high-recall setting. This comparison showed that HybridMouse still managed to identify a higher number of USVs and achieved a significantly higher recall score even in this setting. Lowering the detection threshold also increased the number of false-positive events, resulting in significantly lower precision and overall *F*1 score than HybridMouse.

We further investigated the performance of the models by calculating the global SNR of each tested file and showing that HybridMouse is more robust to lower SNR than DeepSqueak. While the latter performed well and was comparable to the former on files with high SNR, as the levels of SNR dropped, so did the performance of DeepSqueak. Furthermore, pup vocalizations are recorded in a particular setting where the microphone is closer to the recorded pup, and there are very few background noises. Therefore, it was no surprise that these recordings had high SNR and high detection rates by all models.

The high time-resolution and robustness of HybridMouse were achieved by two means. The first one was through the data augmentation methods that we applied to the training data. We added random horizontal and vertical lines, simulating consistent humming and burst noises, respectively. The data augmentation has significantly improved the model by lowering its false-positive rate. The second method was implemented within the model architecture by evaluating each timestep in the contexts of its neighboring timesteps in two levels. The first level was during the CNN layers, where each timestep was fed into the model with its 25 preceding and following time steps. The second layer was in the BiLSTM layers, where each timestep was evaluated in the context of its neighboring 330 timesteps. We also tested other model configurations with different lengths of neighboring timesteps and found that the abovementioned parameters are optimal for maximal accuracy and manageable complexity.

However, this meant that the spectral maps had to be compressed to reduce model complexity. This compression was made by extracting Mel-spectrogram with 124 filter banks, where the lower frequencies were represented with higher resolution than the higher frequencies. In addition, the model was trained on data that was only labeled in the time domain and not the frequency domain. The combined effect of these two properties resulted in a model with high temporal accuracy but lower spectral accuracy, specifically when estimating the upper boundaries of the USVs. This limitation should be taken into account when using HybridMouse.

Another limitation is that the software does not characterize the USV structure. Our model's main goal is to extract USVs even in harsh recording settings. Once these USVs are extracted, the users may proceed with the analysis independently, using any number of available methods for clustering, Classification, and syntax analysis on the extracted USVs.

In summary, HybridMouse is a powerful tool for automatic identification and extraction of mouse ultrasonic vocalizations in variable recording conditions. It may be used for high-throughput detection of murine social vocalizations.

## Data Availability

Publicly available datasets were analyzed in this study. This data can be found at: https://mousetube.pasteur.fr/; https://github.com/gutzcha/HybridMuse.

## Ethics Statement

The animal study was reviewed and approved by Institutional Animal Care and Use Committee, University of Haifa.

## Author Contributions

YG: conceptualization, methodology, software, validation, formal analysis, investigation, data curation, writing, and visualization. KB: conceptualization and writing–review and editing. SN: conceptualization, resources, data curation, writing–review and editing, and project administration. LC: conceptualization and data curation. YH-O: conceptualization, supervision, and writing–review and editing. SW: conceptualization, writing–review and editing, supervision, and funding acquisition. All authors contributed to the article and approved the submitted version.

## Funding

This study was supported by ISF-NSFC joint research program (Grant No. 3459/20 to SW), the Israel Science Foundation (ISF Grants No. 1350/12, 1361/17 to SW, and 258/17 to LC), the Ministry of Science, Technology and Space of Israel (Grant No. 3-12068 to SW), and the United States-Israel Binational Science Foundation (BSF Grant No. 2019186 to SW).

## Conflict of Interest

The authors declare that the research was conducted in the absence of any commercial or financial relationships that could be construed as a potential conflict of interest.

## Publisher's Note

All claims expressed in this article are solely those of the authors and do not necessarily represent those of their affiliated organizations, or those of the publisher, the editors and the reviewers. Any product that may be evaluated in this article, or claim that may be made by its manufacturer, is not guaranteed or endorsed by the publisher.
