# Supplementary material for: HybridMouse: A Hybrid Convolutional-Recurrent Neural Network-Based Model for Identification of Mouse Ultrasonic Vocalizations
Source: Front Behav Neurosci. 2022 Jan 25;15:810590. doi: 10.3389/fnbeh.2021.810590 (PMC8823244; doi:10.3389/fnbeh.2021.810590)
Supplement: Supplementary file 1 [file Data_Sheet_1.PDF]

Supplementary Material

1 Supplementary Tables

Supplementary Table 1. Test data source and details

The models were tested using audio recordings downloaded from the mouseTube database. The file numbers and order is preserved for the following tables.

File	Laboratory	Experiment Name	File Name	Paradigm	Subject Number	Recording Day
**1**	Duke University Medical Center	Social context comparisons	3BB89473_AFCompStim2014-01-08_0000003	AF	1	3
**2**	Duke University Medical Center	Social context comparisons	3BB89473_AMCompStim2014-01-22_0000001	AM	1	1
**3**	Duke University Medical Center	Social context comparisons	3BB89473_AMCompStim2014-01-24_0000003	AM	1	3
**4**	Duke University Medical Center	Social context comparisons	3BB89482_AMCompStim2014-01-22_0000001	AM	2	1
**5**	Duke University Medical Center	Social context comparisons	BB89473_FECompStim2014-01-15_0000001	FE	1	1
**6**	Duke University Medical Center	Social context comparisons	3BB89482_FECompStim2014-01-15_0000001	FE	2	1
**7**	Washington University	Pup USV Day 3-14 Monitoring	TCS1110	Pups	1	1
**8**	Washington University	Pup USV Day 3-14 Monitoring	TCS1137	Pups	2	1
**9**	Washington University	Pup USV Day 3-14 Monitoring	TCS1158	Pups	3	1

Supplementary Table 2 Comparing detection parameters of the three models for all the tested files

Comparing the total numbers of detected USVs, true positives, false positives, and false negatives detected by the HybridMouse model and the DeepSqueak model with two different settings; balanced recall and high recall for all the tested files.

File*	USV#	Detected			True Positive			False Positive			False Negative		
		HM	DS_B	DS_H	HM	DS_B	DS_H	HM	DS_B	DS_H	HM	DS_B	DS_H
1	457	365	294	338	355	286	319	10	8	19	102	171	138
2	32	43	18	39	32	6	7	11	12	32	0	26	25
3	75	82	64	87	69	53	56	13	11	31	6	22	19
4	471	465	420	456	461	416	436	4	4	20	10	55	35
5	453	445	244	329	416	217	269	29	27	60	37	236	184
6	638	681	543	622	630	512	543	51	31	79	8	126	95
7	92	91	77	77	91	77	77	0	0	0	1	15	15
8	70	69	61	61	69	60	60	0	1	1	1	10	10
9	105	102	95	95	102	95	95	0	0	0	3	10	10

* For file details, see Supplementary Table 1
# Total number of USV in the file

Supplementary Table 3 Comparing detection scores of the three models for all the tested files

Comparing recall, precision, and F1 scores of the HybridMouse model to the DeepSqueak model with two different settings; balanced recall and high recall for all the tested files.

File*	Recall			Precission			F1 Score		
	HM	DS_B	DS_H	HM	DS_B	DS_H	HM	DS_B	DS_H
1	0.78	0.63	0.70	0.97	0.97	0.94	0.86	0.76	0.80
2	1.00	0.19	0.22	0.74	0.33	0.18	0.85	0.24	0.20
3	0.92	0.71	0.75	0.84	0.83	0.64	0.88	0.76	0.69
4	0.98	0.88	0.93	0.99	0.99	0.96	0.99	0.93	0.94
5	0.92	0.48	0.59	0.93	0.89	0.82	0.93	0.62	0.69
6	0.99	0.80	0.85	0.93	0.94	0.87	0.96	0.87	0.86
7	0.99	0.84	0.84	1.00	1.00	1.00	0.99	0.91	0.91
8	0.99	0.86	0.86	1.00	0.98	0.98	0.99	0.92	0.92
9	0.97	0.90	0.90	1.00	1.00	1.00	0.99	0.95	0.95

* For file details, see Supplementary Table 1

2 Supplementary Figures

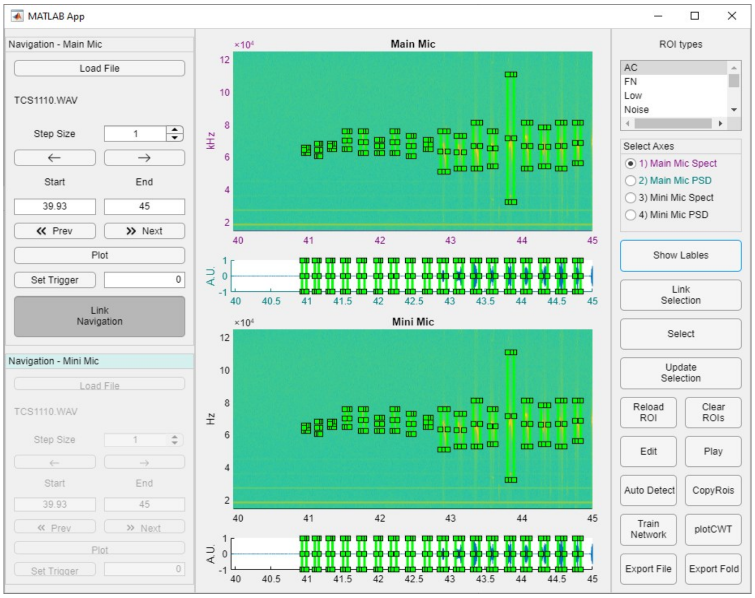

Supplementary Figure 1. The HybridMouse app main window. The HybridMouse software a complete manual and example audio files will be available to download from our Github repository

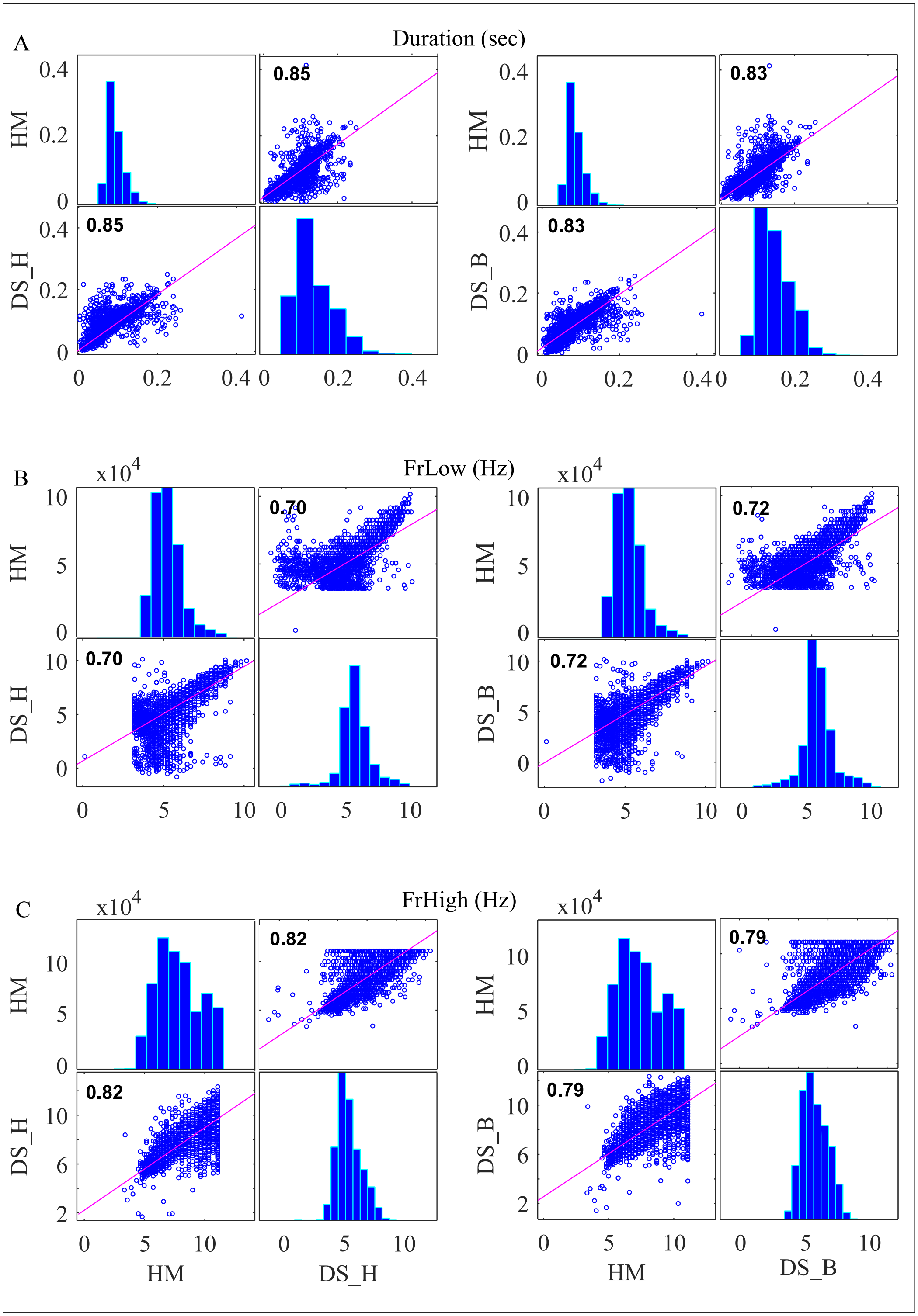

Supplementary Figure 2. Correlation of (A) Duration, (B) lower frequency boundaries (FrLow), and (C) higher frequency boundaries (FrHigh) of the detected USVs estimated by HybridMouse (HM) and DeepSqueak in high recall (DS_H) and balanced (DS_B) recall settings. Plots in the upper left and lower right corners show the distributions in each panel. The plots in the upper right and

lower left corners show the correlation (Pearson's linear correlation coefficient) between the metrics estimated by the different models.

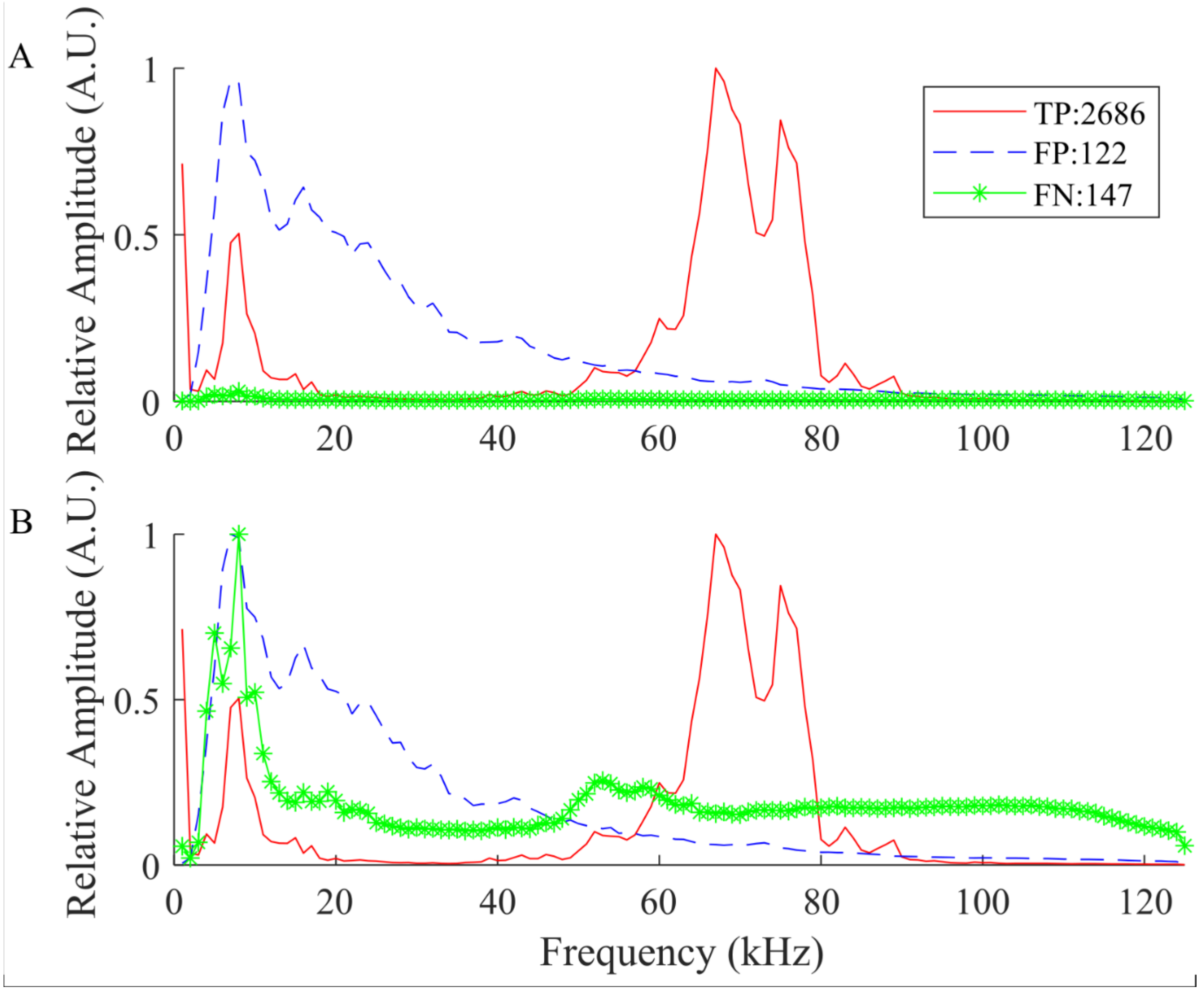

Supplementary Figure 3 Comparing spectral properties of detected USVs and missed USVs

The power spectral density (PSD) of the detected USVs (TP, solid red line), the mislabeled noise segments (FP, dashed blue line), and the missed USVs (FN, solid green line with asterisks) is shown. The numbers in the legend indicate the number of events for each type. There are no USVs emitted at frequencies lower than 30kHz; therefore, we can safely assume that high values in this range are produced by noise, while high values in the higher frequencies are likely to be produced by USVs (signal). (A) The PSD values were scaled uniformly by dividing them by the maximal value al all types. The relative amplitude of the missed USVs is low compared to the other types. (B) The PSD values were scaled independently for each type, showing relatively high values in the lower frequencies, indicating that the missed USVs have low SNR.
